# Individual Differences in Performance Speed Are Associated With a Positivity/Negativity Bias. An ERP and Behavioral Study

**DOI:** 10.3389/fnbeh.2020.00007

**Published:** 2020-01-31

**Authors:** Naira A. Taroyan, Beatrice Butnicu, Antonia Ypsilanti, Paul G. Overton

**Affiliations:** ^1^Heart of the Campus, Collegiate Crescent Campus, Sheffield Hallam University, Sheffield, United Kingdom; ^2^Department of Psychology, The University of Sheffield, Sheffield, United Kingdom

**Keywords:** positivity bias, ERP, LDT, individual differences, emotion regulation, cognitive abilities

## Abstract

There is a current dispute over the origins, incidence, and development of Positivity Bias, i.e., preferential processing of positive relative to negative information. We addressed this question using a multi-method technique of behavioral, psychometric and event-related potential (ERP) measures in a lexical decision task (LDT). Twenty-four university students (11 female) participated (age range 18–26), but four were omitted owing to data issues. Participants were classified as Positivity Biased (PB) if their LDT responses to positive words were faster than negative words, and vice versa for those classified as Negativity Biased (NB), leading to a group of 11 PB participants and a group of 9 NB participants. Interestingly, the PB group was significantly faster overall than the NB group and had significantly shorter P2 component ERP latencies in the left occipital region. Furthermore, the PB group had significantly higher scores for expressive suppression (ES), together with higher scores for Crystallized Knowledge and for cognitive reappraisal (CR). These results suggest that around 55% of the students had Positivity Bias, and these were more efficient in processing information and had better emotion regulation abilities than those with a Negativity Bias.

## Introduction

Human emotions are as diverse as the number of people in the world (Kagan, 2007), with various theories attempting to explain what constitutes an emotion. According to the duality of mind model, a direct evaluation of external stimuli is carried out by an automatic evaluating system (AES), and more effortful linguistic processing and cognitive reappraisal (CR) by a reflective evaluating system (Jarymowicz and Imbir, 2015). Thus, the origin of the emotional state can modulate cognitive processing (Imbir et al., 2016) and CR is important in emotion regulation whereby an individual can re-evaluate their initial response by reappraising the stimulus to change the meaning of the event, which subsequently can contribute to improved mental health (Gross and John, 2003).

A “Positivity Bias” (PB) is defined in terms of preferential (that is, faster) processing of stimuli with positive emotional salience (e.g., Leppänen et al., 2003) and it has been widely documented in older adults (e.g., Carstensen et al., 2000). However, it is unclear whether there is a bias in the processing of emotionally salient information in younger adults. While some studies in younger adults report a preference for positive information processing, others show the opposite pattern. For example, positive facial expressions (Leppänen and Hietanen, 2004) and words (Stenberg et al., 1998) can be recognized or categorized faster than negative stimuli in younger adults. On the other hand, faster performance speed (e.g., Kousta et al., 2009) and greater early brain activation (Zhang et al., 2014) to negative words have also been reported.

The reason for these contradictory findings is unclear, however, one possibility is that previous studies have erroneously ignored the presence of individual differences in the processing of emotionally salient information, and the effect that the presence of those differences might have on the outcome of the studies. It has been suggested recently that there may be genetically pre-determined individual differences in positivity offset (neutral stimuli are viewed more positively) and negativity bias (Ito and Cacioppo, 2005; Ashare et al., 2013). The authors suggested that genotype/gender interactions may predetermine altered serotonin transmission in some brain structures, including the amygdala, associated with negative affect. Thus, it is possible that negativity/positivity bias is a genotype predetermined innate feature that is accompanied in some cases with deficits in amygdala inhibitory (i.e., decreased activation) function, particularly in low cognitive functioning adults (Winecoff et al., 2011). Diminished amygdala responses are related to reappraisal aimed to decrease negative affect (see also Ochsner et al., 2004), whereas expressive suppression (ES) of negative information is related to slower responses in the prefrontal cortex and increased activation in the amygdala (Goldin et al., 2008). Thus, higher cognitive abilities and improved executive function contribute to a stronger positivity effect (Mather and Knight, 2005) and better startle response inhibition in older adults (Gyurak et al., 2009). It has also been reported that these features may be the result of not only biological but also environmental and cultural factors. Thus, as suggested by Borkenau et al. (2010), life circumstances and individual differences in temperament, such as extraversion, could predict predisposition for negative and positive trait affect. Using a lexical decision task (LDT), extraversion and approach temperament was shown to be associated with faster reaction times (RTs) to pleasant compared to neutral and negative words. It was claimed in another LDT study that individual differences in vocabulary knowledge were associated with performance speed in 819 native English speaking university students (Yap et al., 2015). Additionally, recent research showed that a left-lateralized decrease in electroencephalography (EEG) alpha band and consequently right-lateralized cortical activation was linked to a positivity effect, whereas the opposite pattern of cortical activation was related to a negativity effect (Mueller and Kuchinke, 2016).

Thus, recent research suggests that positivity/negativity biases may be a result of various individual differences in cognitive processing, and hence the seemingly contradictory findings about those biases in younger adults may simply relate to unplanned selection bias in the constitution of the sample used in the studies. To further assess the potential importance of individual differences in emotional biases in younger adults, in the present study we used an LDT—a novel approach to this issue. The majority of the literature on positivity bias reports research using mostly images and facial expressions, autobiographical memory for positive and negative events, health messages and explicit decision-making paradigms (for review, see Reed et al., 2014). To the best of our knowledge, no research on individual differences in this phenomenon has been performed using implicit reading emotion processing tasks, such as the LDT. Having an implicit task like the LDT is likely to be advantageous in the context of individual differences in emotional biases as a recent meta-analysis suggested that manipulations in explicit experimental tasks and conditions may hamper the presence of a positivity bias (Reed et al., 2014).

In the context of a preference for positive or negative information processing using an LDT, one useful approach has been to employ event-related potentials (ERPs). For example, larger ERPs were recorded overall to emotion words compared to neutral words regardless valence (e.g., Bayer et al., 2012), with more inconsistent results of later ERPs being larger to negative compared to positive words (e.g., Kanske and Kotz, 2007), or vice versa (e.g., Zhang et al., 2014). The ERP technology, which we use in the present study, offers high temporal resolution recording of brain activation which makes it possible to examine cognitive components that are relevant to emotion regulation.

In the current study, we aimed to explore the effect of individual differences in cognitive abilities and emotion regulation (both reported to affect performance in emotion word processing), on positivity bias using an LDT/ ERP paradigm in young adults. Examining positivity effects in a group of young adults, while controlling for general cognitive ability is important in understanding the underpinnings of the previous contradictory findings in this population. The present study also contributes significantly and extends the existing literature on positivity effects as we question whether other individual differences (in addition to cognitive), such as the general speed of task processing, might be related to the positivity/negativity effect too. The combination of these measures is aimed to determine whether there is a positivity (or negativity) bias in the behavioral performance of our participants depending on their individual differences and whether these differences are related to brain activation indices of their performance. Thus, this study provides for the first time combined behavioral and ERP research on emotion word processing and the effect of individual differences, including cognitive abilities, emotion regulation, performance speed and accuracy, on positivity/negativity bias. We predict that some young adults will show a positivity bias and some young adults will show a negativity bias, indicated both in terms of their word/non-word discrimination performance and electrophysiological indices of cognitive processing.

## Materials and Methods

### Participants

Twenty-four Sheffield Hallam University students (11 females) between the ages of 18 and 26 (*M* = 20.54; *SD* = 2.19) were recruited in exchange for credits towards their undergraduate psychology degree. All participants were right-handed native English speakers, with normal or corrected-to-normal vision, no history of neurological disorders, traumatic brain injuries, learning disabilities or any medication taken at the time of participation. They were screened for depression and had low scores in the range of 0–11 (*M* = 4.36; *SD* = 3.97). Four participants were removed *post hoc* due to insufficient data or significantly noisy ERP waveforms. They had no other deviations from the group average on any other measures used here. The study was approved by the local ethics committee of the Psychology Department at Sheffield Hallam University, and written informed consent was obtained from all participants before the testing commenced.

### Cognitive Ability

Fluid intelligence (FI) was tested by the block design subsection of the *Wechsler Abbreviated Scale of Intelligence–Second Edition* (WASI-II; Wechsler, 2011). The task involved a number of blocks with various color patterns on each side that had to be rearranged to match a presented pattern shape. The series of shapes increased in difficulty from four block patterns with a 60 s limit to nine block patterns with a 120 s limit. Scores were determined by accuracy as well as speed of completion.

Crystallized knowledge (CK) was tested by the *Mill Hill Vocabulary Scale* (Raven, 1958). The test involved two sections, each with a list of 44 words progressing in difficulty (e.g., bread vs. abnegation) and required participants to provide a brief explanation in writing of the words’ meanings. The second task required the respondent to choose for each word another one closest in meaning from six given alternatives.

### Emotion Regulation

Emotion regulation was measured on two dimensions, namely CR and ES using Gross and John (2003) *Emotion Regulation Questionnaire* (ERQ). The test involved 10 self-report items, six to assess CR and four to assess ES, which could be answered on a 7-point Likert-type scale ranging from 1 (strongly disagree) to 7 (strongly agree). The coefficients for CR (Cronbach’s *α* = 0.80) and ES (Cronbach’s *α* = 0.74) indicated the internal consistency of test scores on both subscales (e.g., Spaapen et al., 2014).

### Depression

To ensure that measures used and the performance of our participants were not affected by underlying depression symptoms *Beck’s Depression Inventory-I* (BDI; Beck et al., 1961) was used. This test was a self-report inventory consisting of 20-one items asking about a person’s mood and feelings experienced in the last week. Respondents answered the items choosing from four possible responses, ranging from 0 to 3 in intensity [e.g., (0) “I do not feel sad”; (3) “I am so sad or unhappy that I can’t stand it”; Cronbach’s *α* = 0.74; Zhang et al., 1990].

### LDT and Stimuli

The LDT was similar to the task used by Taroyan and Nicolson (2009) and see also Wimmer et al. (2002). One-hundred and thirty-five regular English nouns and 135 pseudowords were presented one at a time in random order. The English nouns were further divided into 45 positive, 45 negative and 45 neutral words, resulting in four main conditions: PosW, NegW, NeuW and Pseudowords (PW). The words were selected and matched for emotional and linguistic characteristics from the Affective Norms for English Words (ANEW; Bradley and Lang, 1999), while pseudowords were selected from the ARC Nonword Database (Rastle et al., 2002) and matched for the number of letters as well as bigram frequency, as differences in the latter were shown to have effects on early ERPs (Hauk et al., 2006).

Positive words were selected based on high valence (*M* = 7.69; *SD* = 0.53), negative words on low valence (*M* = 2.29; *SD* = 0.55), and neutral words closer to the median value (*M* = 4.96; *SD* = 0.43). Both positive and negative words were selected from items with high arousal values, while neutral words had significantly lower arousal values. Dominance was not used explicitly to select items, as it is a less frequently used emotional dimension in word processing studies, however, it was shown to correlate highly with valence (Warriner et al., 2013), as it did in the present study (*r* = 0.87; *p* < 0.001).

Both words and pseudowords contained 5–9 letters (*M* = 6.25; *SD* = 1.04) displayed on a 20″ PC monitor in black, lower case, bold Times New Roman font on a light gray background at 60 cm viewing distance. The task was designed and behavioral data (performance speed and accuracy) recorded in E-Prime.

### Procedure

Participants received instructions and information sheets in preparation for the study. They were asked to be well-rested on the day, to avoid consuming caffeine or nicotine and to have their hair freshly washed and devoid of any hair products as these may interfere with the electrode impedances. After written informed consent was given, participants completed the BDI, ERQ and Mill Hill. The EEG recording caps were simultaneously applied and impedances adjusted at this time. Depending on participants’ writing speed and ease of electrode preparation, this part of the testing lasted around 20 min. Participants were given a short break, after which the WASI-II was administered, followed by another short break in preparation for the LDT.

Participants were seated in a comfortable chair in front of a PC monitor in an electrically shielded, light-attenuated room. The task required them to press the upper button on a response box with their right index finger if the displayed stimulus was a word and the lower button with their middle finger if it was a non-word. There were 270 trials in the LDT (135 words and 135 PW) consisting of a fixation period (a small black cross presented in the center of the screen for 1,000 ms), followed by the stimulus for 2,000 ms, and a blank screen for another 2,000 ms. The task lasted about 15 min.

Participants were asked to focus on the fixation cross and avoid any eye, head, and general body movements during the stimulus display. A few min practice to confirm participants understood the task was provided before actual testing. The task was launched by the experimenter from the adjacent room, and performance was monitored through a paired monitor. The whole experiment lasted about 40–50 min.

### Data Acquisition

The EEG was recorded *via* a 64 channel waveguard™ original cap from ANT Neuro BV with a vertex reference. The waveguard cap consisted of Ag/AgCl electrodes adjusted individually until impedances reached values below 10 kΩ. The waveguard cap was connected to the asalab™ high-resolution active shielding amplifier with high input impedance. The EEG was recorded within a range of 0.016 Hz–200 Hz through ASA 4.7.8 software and stored for further analysis. Response accuracy RTs for each stimulus condition were also recorded and stored through E-Prime, while the BDI, ERQ, Mill Hill and WASI-II paper materials were stored along with written informed consent in a secure location.

### Data Filtering and Artifact Removal

The EEG data were analyzed with ASA 10 software and were manually bandpass filtered within 0.03–40 Hz with a 24 slope (dB/oct) filter steepness (0.016–256 Hz) to avoid 50–60 Hz noise interference from potential electrical sources. The waveforms were divided into epoch events starting 100 ms prior to stimulus onset and lasting 1,000 ms post-stimulus onset. A few samples of artifacts were manually detected to ensure the software launched an accurate automatic artifact detection afterward. Artifact detection was employed by manually setting a range between −70 μV and 70 μV with DC correction, followed by further visual investigation of residual artifacts outside of the established range and adjusted accordingly. Automatic artifact correction was then performed with a threshold of ±70 μV.

### Data Analysis

The regions of interest (ROI) and electrode locations used for further analysis in this study were occipital (O), parieto-occipital (PO) and parietal (P) channels selected due to their relevance in visual word recognition and formation (e.g., González-Villar et al., 2014; D’Angiulli et al., 2015). Visual inspection indicated that activation in these areas had a similar pattern and had the largest and clearest ERP peaks.

The behavioral and EEG data underwent additional processing and analysis using E-Prime and ASA 10, respectively. Mean RTs and number of correct responses and errors were processed for each participant. Test scores for the BDI, ERQ, WASI-II and Mill Hill were calculated manually.

**Figure 1:**
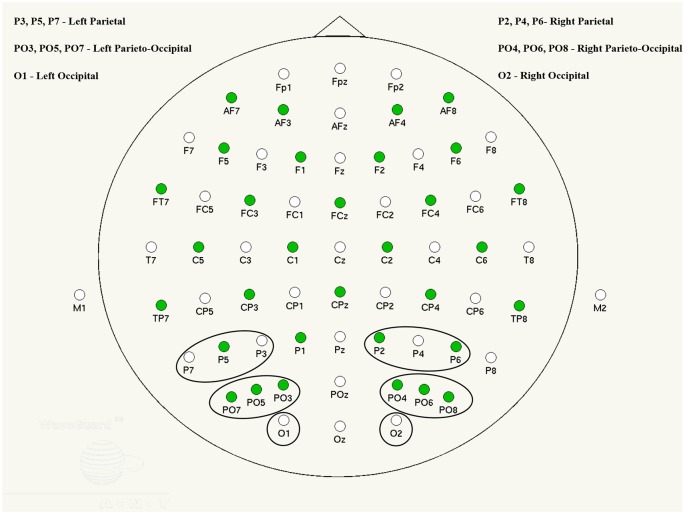
ANT waveguard 64 electrode cap layout and channel groups selected for analysis.

ERPs were derived from condition-specific EEG epochs accompanied by a correct response and computed within 100 ms prior to and 1,000 ms after the stimulus onset. These events were then averaged and baseline corrected across individual electrodes and areas of interest in the left and right hemisphere (see Figure 1).

Grand averages were determined separately for each group (for a description, see “Results” section) and in each condition. ERP components were visually analyzed in group average and individual participant waveforms, in order to determine characteristic peaks and their latency time-windows. Thus, three distinct components were found: P1 (~110 ms), N1 (~170 ms) and P2 (~270 ms), with corresponding group average time windows of 90–120, 140–190 and 200–300 ms. These components were also reported in previous literature to be early (P1) and later subsequent emotion processing correlates (Prete et al., 2018). A late P300 was identified in some individuals, however it was not present in all participants and, therefore, was excluded from further analysis. Each participant’s ERP peak amplitudes were computed within the time windows established in the group average ERPs and automatically measured at the peak maximum (e.g., Taroyan, 2015). Peak amplitudes and latencies for all single channels were averaged into side-specific area groups (O, PO and P, for left and right hemisphere separately; see Figure 1) in order to optimize signal to noise ratio and increase statistical power (Oken and Chiappa, 1986).

### Statistical Analysis

For behavioral analysis, two factors mixed measures analysis of variance (ANOVA) were carried out separately for mean RTs and errors with the within-subjects factor Condition (PosW, NegW, NeuW and PW) and between-subjects factor Group (PB and negativity biased (NB), for a description, see “Results” section). Independent *t*-tests were carried across all measures with the between-subjects factor. Pearson correlations and planned *t*-tests were carried out across all measures, particularly for emotion regulation and its relation to RTs across conditions, as well as comparing between the PB and NB groups.

For the ERP analysis, the resulting average amplitude and latency values of P1, N1 and P2 ERP components for each participant were checked for outliers greater than three standard deviations using Tabachnick and Fidel’s (1996) method and subjected to a four-factor mixed measures ANOVA with Group (PB and NB) as the between-subjects factor and three within-subjects factors: condition (PosW, NegW, NeuW and PW), Area (O, PO and P) and Hemisphere (Left and Right). When Mauchly’s test of sphericity suggested significant non-sphericity, the Greenhouse-Geisser correction was selected. When main effect interactions were found, planned *t*-tests were carried out to further investigate.

## Results

### Behavioral Data

**Figure 2:**
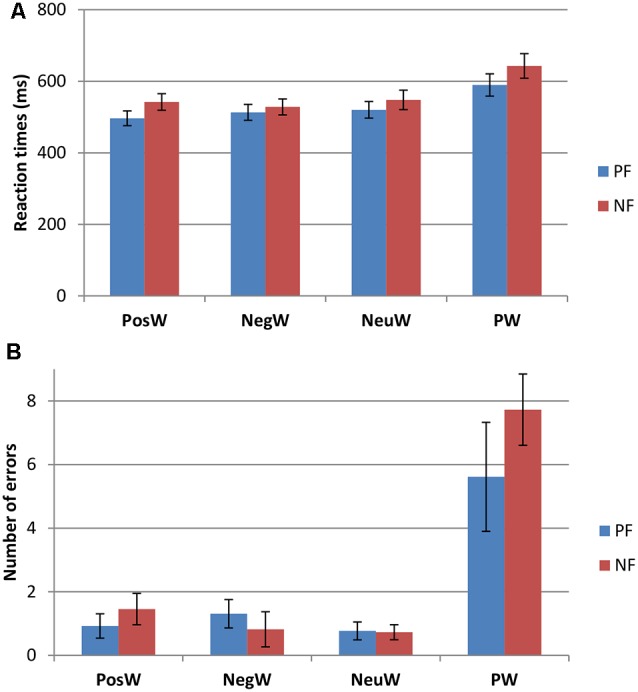
Behavioral data for the Positivity Biased (PB) and Negativity Biased (NB) groups (mean ± SE) for **(A)** reaction times (RTs) and **(B)** performance accuracy.

Participants were divided into two groups, Positivity Bias (PB) and Negativity Bias (NB), with the operational definition of PB being that the mean response speed was faster for positive words than for negative words, and the NB group faster for negative words than positive words. This led to two groups, whereby nine people (five female), with a mean age of 21.11 years (*SD* = 2.47), were faster to negative words as compared to positive, and 11 people (five female), with a mean age of 20.45 years (*SD* = 1.81) were faster to positive words. Further analysis revealed significant differences in RTs in the PosW compared to the NegW condition for each group, in that the PB group had significantly faster RTs to positive than to negative words (*t*_(12)_ = −6.951, *p* < 0.001), and the NB group had significantly faster RTs to negative than to positive words (*t*_(10)_ = 2.454, *p* < 0.034). These differences can be observed in Figure 2. The groups were not automatically called positivity and NB at this stage as the further analysis was necessary and RTs needed to be compared and related to their respective ERP activation.

Two factors mixed measures ANOVAs for mean RTs showed only a main effect of Condition (*F*_(3,54)_ = 33.526, *p* < 0.001). Pairwise comparisons revealed that there were significantly longer RTs to pseudowords than to any word condition (see Figure 2), which was to be expected (e.g., Taroyan and Nicolson, 2009). There were significantly longer RTs to neutral words than to positive words as well (*p* < 0.04). There were no significant group or interaction effects. The same analysis was carried out for the errors in this LDT task and again only Condition was significant with higher scores in PW condition compared to others (*F*_(3,54)_ = 27.675, *p* < 0.001).

No significant differences were found between the groups on any of the cognitive measures, the closest to significance being on CK, whereby the PB Group (*M* = 51.09, *SD* = 6.76) appeared to have higher CK than the NB Group (*M* = 46, *SD* = 4.47; *t*_(18)_ = 2.016, *p* = 0.60).

However, there was a significant difference between the groups in emotion regulation, particularly ES whereby the PB Group (*M* = 14.91, *SD* = 4.23) suppressed significantly more than the NB Group (*M* = 10.44, *SD* = 2.96; *t*_(18)_ = 2.67, *p* < 0.02). The scores on CR were also slightly higher in the PB (*M* = 29.45) compared to the NB (*M* = 26.49) group.

In summary, RTs and errors were larger for pseudowords than other conditions, and RTs were longer to NeuW than to PosW condition. Some participants had longer RTs to positive words than negative words and some had longer RTs to negative words than to positive words. The PB group had faster RTs overall and higher scores in Crystallized Knowledge, ES (significantly) and CR. Hence, the PB group shows more efficient processing, not only in terms of emotion regulation and cognitive abilities but also in terms of processing speed.

### ERP Data

**Figure 3:**
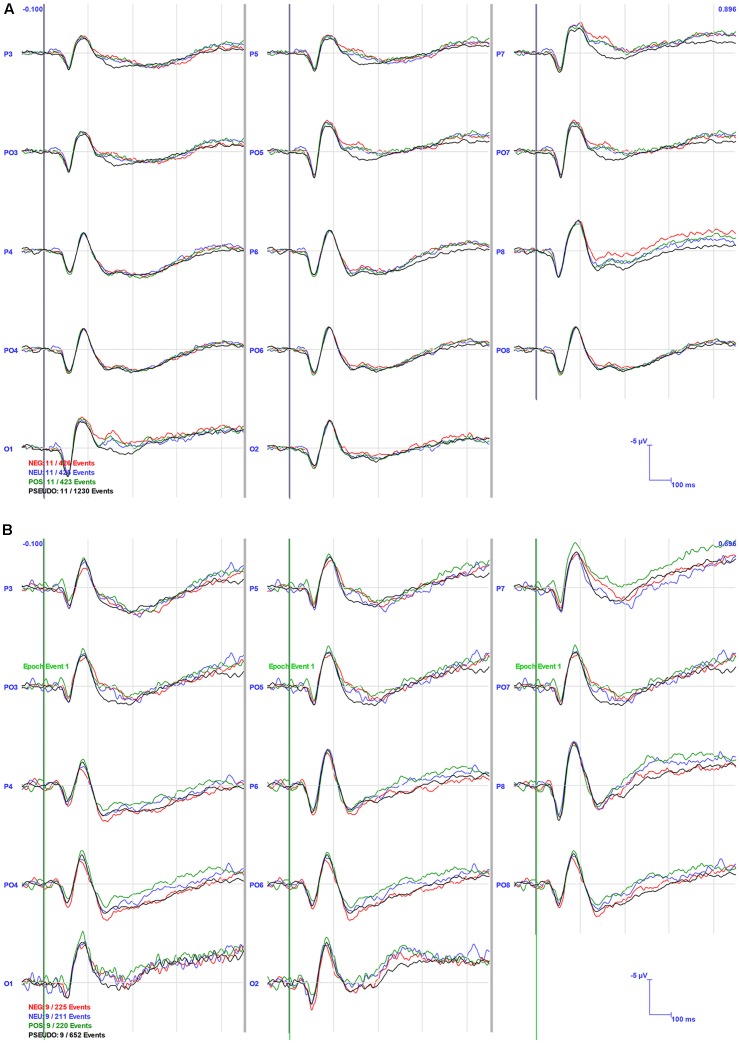
Grand Averaged event-related potentials (ERPs) for the PB **(A)** and NB **(B)** Groups in selected channel groups. The bold vertical lines indicate the stimulus onset at 0 ms. The channel locations are specified at the start of the waveforms and conditions are indicated in color in the left bottom corner of the graph.

Visual inspection of the grand averages (Figure 3) suggested larger amplitude ERP peaks in the NB compared to the PB group, e.g., N1 in area P6 was almost twice as large in the NB (−5.09 mV) compared to the PB (−2.85 mV) group averages.

### P1 Component

Statistical analysis of the ERP data showed no significant differences for the P1 amplitude, however, there was a significant main effect of Area for P1 latency (*F*_(2,54)_ = 8.031, *p* < 0.001). Bonferroni adjusted pairwise comparisons revealed that there was no significant difference in latency between the P and PO areas but significantly longer P1 latencies in the PO (*M* = 106.65, *SD* = 1.8) and P (*M* = 106.97, *SD* = 1.5) compared to the O (*M* = 103.96, *SD* = 1.74) area. No significant group effect was found. There was a significant interaction effect between Group and Side (*F*_(1,54)_ = 7.092, *p* < 0.02), however planned *t*-tests showed significant difference (*t*_(18)_ = 2.914, *p* < 0.02) for PW only, whereby the PB group (*M* = 108.82, *SD* = 11.95) had longer latencies than the NB group (*M* = 96.94, *SD* = 5.71) in the right occipital area. There was a significant interaction effect found between Condition and Side (*F*_(3,54)_ = 3.841, *p* < 0.02), however, no significant differences were found in the pairwise comparisons.

### N1 Component

**Figure 4:**
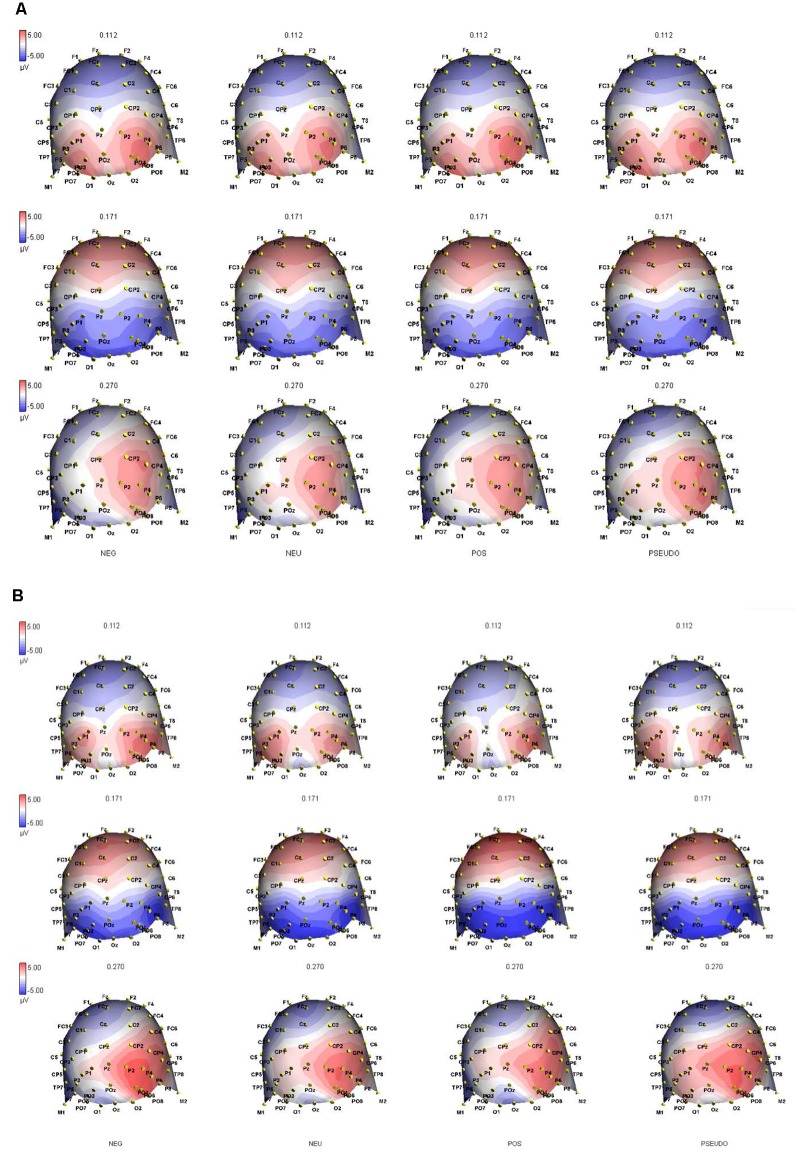
Topographic ERP maps capture the distribution of brain electrical activation at peak latencies of the P1 (at 112 ms), N1 (at 171 ms) and N2 (at 270 ms) components in **(A)** the PB and **(B)** NB groups and all four conditions indicated at the bottom of the graphs. The black symbols on the maps represent the individual channels in 64 channel ANT waveguard cap.

For N1 amplitude, there was a main effect of Area (*F*_(2,36)_ = 6.574, *p* < 0.01), whereby parietal areas (*M* = −4.76, *SE* = 0.69) had significantly higher amplitudes than occipital areas (*M* = −6.35, *SE* = 0.81). No significant interactions or group effects were found, although, as can be seen in Figures 3, 4, N1 amplitude in the right parietal area (channels P4, P6, and P8) was larger, particularly for the NB group, as compared to the left hemisphere (LH; channels P3, P5 and P7).

For N1 latency, there was a significant main effect of Area again (*F*_(2,36)_ = 8.059, *p* < 0.001) whereby the PO (*M* = 170.99, *SE* = 3.09) and P areas (*M* = 174.73, *SE* = 2.63) had significantly longer latencies than the O areas (*M* = 167.97, *SE* = 2.85). There was a significant Condition × Area (*F*_(6,108)_ = 3.009, *p* < 0.01) and Condition × Area × Group (*F*_(6,108)_ = 2.327, *p* < 0.04) interaction, however follow up *t*-tests did not reveal any significant differences thereafter.

### P2 Component

There was a significant main effect of Side (*F*_(1,18)_ = 13.832, *p* < 0.003) where the right hemisphere (*M* = 3.91, *SE* = 0.72) had significantly higher amplitudes overall as compared to the LH (*M* = 1.32, *SE* = 0.49). There was also a significant main effect of Area (*F*_(2,36)_ = 5.559, *p* < 0.01) where parietal areas (*M* = 2.96, *SE* = 0.49) had significantly higher amplitudes than did occipital areas (*M* = 2.05, *SE* = 0.56). No significant Group effects were found.

For P2 latency, there was a significant main effect of Area (*F*_(2,36)_ = 7.416, *p* < 0.02), where parietal areas (*M* = 272.38, *SE* = 2.91) had significantly longer latencies than did occipital areas (*M* = 260.14, *SE* = 5.42). There were significant interaction effects: Condition × Side × Area (*F*_(6,108)_ = 3.425, *p* < 0.005) and Side × Area × Group (*F*_(2,36)_ = 4.504, *p* < 0.02). For the first one, pairwise comparisons revealed a significant difference between the PosW and NegW word conditions in the parietal area (*t*_(19)_ = −2.299, *p* < 0.03), whereby there were significantly longer latencies in the left parietal area when visualizing negative words (*M* = 276.59, *SD* = 24.54) than positive words (*M* = 260.02, *SD* = 31.89). Interestingly, for the Side*Area*Group interaction effect, One-Way ANOVA showed that the PB group had a significantly shorter latency to positive words (*M* = 236.45, *SD* = 23.97) than did the NB Group (*M* = 266.84, *SD* = 33.88) in the left occipital area (*F*_(1,19)_ = 5.512, *p* < 0.04). Although no significant Group main effect was found, this ERP interaction effect seems to echo the positivity bias found in our behavioral data.

In summary, the main ERP results show that the amplitude and latency of the ERP peaks were overall larger in parietal areas compared to occipital, as well as in the right hemisphere compared to the LH. The latency of P2 was faster to PosW than to NegW in the LP area. In terms of Group differences, the PB group had shorter P2 latencies to positive words than to negative words in the LO area. This result, together with shorter RTs found to PosW, indicates more efficient cognitive processing in our PB group.

## Discussion

This study was designed to investigate the role of individual differences in emotion regulation, cognitive abilities and performance efficiency in positivity/negativity bias using a novel combined LDT/ERP paradigm. In terms of emotion words processing our behavioral results showed two distinct groups, with the PB being faster in response to positive words and the NB to negative words. Additionally, the PB group was faster overall in the LDT task, had higher ES, Crystallized Knowledge and CR scores. These findings were accompanied by more efficient brain activation and shorter ERP response latencies. There were also more general findings, whereby greater brain activation overall was recorded in the right compared to the LH, as well as in parietal regions compared to occipital. These findings corroborate previous reports of right hemisphere dominance in emotion processing (e.g., Patel et al., 2018), and are discussed in more detail below.

First, some of our participants were faster to positive compared to negative words, and others were faster to negative words. The PB group were also faster in their LDT performance overall, i.e., across all conditions, compared to negatively biased people. These differences were also accompanied by better ES, CR, and Crystallized Knowledge values. Our findings replicate previously reported individual differences in positivity bias that depend on their *cognitive abilities*. Thus, as reported by Winecoff et al. (2011), higher functioning individuals are more efficient and show preference for positive information processing compared to low cognitive functioning individuals. We show this effect in a different paradigm using an implicit task of word/nonword discrimination and confirm once more that: (a) emotionally valenced information is more salient and preferred to neutral information as indexed by overall faster responses to positive words compared to neutral; and (b) people with better emotion regulation and cognitive abilities are positively biased, as indexed by our PB group’s faster responses to positive compared to negative words. The additional interesting finding in the current study is that our positively biased participants were not only cognitively more able and had better emotion regulation techniques but they were also more efficient overall in terms of performance speed. Previous research showed individual differences in performance speed depending on higher cognitive abilities, such as vocabulary knowledge (e.g., Yap et al., 2015) and IQ (e.g., Der and Deary, 2018).

There were also previous reports of positivity bias related to better *emotion regulation* ability but these results are mixed, with CR of negative information, on one hand, related to diminished amygdala responses (Ochsner et al., 2004) and ES of negative emotional content, on the other hand, to increased amygdala and slower prefrontal cortex activation (Goldin et al., 2008). In the current study, however, both CR and ES were related to positive information processing bias. This may indicate that these individuals were simply better at regulating emotions, particularly in control of negative words processing, in that they suppressed these, and preferentially processed and estimated more efficiently positive words. Thus, our behavioral results overall show that more *efficient speed of performance* accompanies positivity bias in this study and that is also related to better cognitive and emotion regulation abilities. These findings support research evidence of executive function correlation with better emotion regulation (e.g., Krendl et al., 2009) and positivity effect (Mather and Knight, 2005).

Our behavioral results were supported by changes in *ERP* activation. First, we discuss more general findings in the brain activation data that replicated previously reported findings, namely right hemisphere dominance in emotion processing and seemingly parietal area specialization in our LDT emotion words processing. It has been shown before that emotions are processed more efficiently in the right hemisphere as compared to the LH. Thus, according to the Right Hemisphere Hypothesis (RHH) there is a general dominance of the RH in all kinds of emotions, and emotional reactions may be inappropriate in right-brain damaged patients (Gainotti, 2019). Evidence from lesion (e.g., Adolphs et al., 2003) and transcranial magnetic stimulation (e.g., Pitcher et al., 2008) studies have demonstrated right cortex dominance in facial emotion recognition. However, these findings may be contradictory to the valence hypothesis (VH) that suggests LH dominance for positive information processing and right hemisphere (RH) dominance for negative emotion processing (Baijal and Srinivasan, 2010). However, Prete et al. (2018) argued that these two hypotheses are not mutually exclusive and while their combined behavioral and ERP data showed RH dominance for negative emotions processing, LH dominance in behavioral data and RH superiority in ERP findings were found for positive emotions. According to our ERP results, emotion word processing is *greater in the RH*, as indicated by our later, P2 component amplitude. At the same time, P2 latency was shorter to PosW compared to NegW in the LP area indicating more efficient processing. These two results corroborate the RHH/VH hypotheses discussion on RH/LH involvement in the processing of emotions briefly reported above.

Another general finding was the dominance of the *parietal areas over occipital* in our ERP data. Thus, the amplitude of the early P1 and latency of the N1 components, and both amplitude and latency of the P2 component were larger in parietal areas as compared to occipital areas, together indicating greater effort of processing in this area. The parietal cortex dominance in emotional processing has been shown in previous literature, e.g., functional magnetic resonance imaging (fMRI) studies demonstrated the involvement of the parietal cortical regions in processing of emotional faces among other brain areas, such as prefrontal cortex and amygdala (Fusar-Poli et al., 2009). An ERP study on age differences and emotional modulation showed widespread parietal effects for all valences among young adults (Langeslag and van Strien, 2009).

And finally, an ERP result that has only been found in this study is that the P2 component had a shorter latency in the PB as compared to the NB group to positive words in the left occipital area. The location of this effect somewhat contradicts previously discussed suggestions of right parietal area dominance in emotion word processing. However, the advantage of the high-density ERP technique compared to other neuroimaging methods is that differences in processing can be spotted at earlier processing times and/or lower perceptual cortical areas. Thus, the ERP correlates of positivity bias was present and recorded already in the occipital area. However, it was not present in earlier, P1 and N1 components, which might indicate that evaluation and decision making about the nature of the words, whether positive or negative, takes place at slightly later stages of processing (Eimer and Holmes, 2002). This difference between PB and NB groups is even more interesting in that it is accompanied by behavioral faster responses to positive words in the PB group. We consider this result a novel contribution to the positivity bias literature in that cognitively able and efficient processing behaviorally is supported by efficient ERP activity correlates of a positivity bias. We show for the first time that the LDT paradigm and implicit emotion word processing can delineate differences in both behavioral and electrophysiological brain processes and individual differences in positivity bias.

The limitations of the current study are that the sample was mostly from University students and was small. Additionally, the research was mostly data-driven and division into the groups was based on *post hoc* observation of differences in speed of performance overall and to positive and negative stimuli individually. Future research should explore these same measures in an older sample. Also other variables, such as IQ, introversion/extraversion, vocabulary knowledge and other executive functions, in addition to performance efficiency, rather than only emotion-related cognitive abilities, may affect RTs in LDTs and differ between the groups. Additionally, a combination of this technique with fMRI could show the exact localization of emotion word processing using LDT, as ERPs are known for their temporal rather than spatial resolution.

In conclusion, we have shown that processing emotion words is similar to processing other emotion stimuli used in previous literature and that a positivity bias depends on individual differences not only in cognitive abilities and emotion regulation efficiency but also in performance speed efficiency. We also showed that behaviorally distinct performance patterns are accompanied by differences in ERP correlates of the positivity effect. Thus, we show in this research that the phenomenon is found in a young adult population and we confirm previous claims of positivity bias not only being dependent on cognitive and emotion regulation efficiency but also on speed of behavioral and electrophysiological activation.

## Data Availability Statement

The datasets generated for this study are available on request to the corresponding author.

## Ethics Statement

All procedures performed in this study involved human participants and were in accordance with the ethical standards of the Sheffield Hallam University research committee and with the 1964 Helsinki declaration and its later amendments or comparable ethical standards. Informed consent was obtained from all individual participants included in the study.

## Author Contributions

NT and BB have collected and analyzed the data and have written the majority of the manuscript. AY has provided ideological and organizational aspects of preparing the research project. PO has helped with structuring and editing the manuscript.

## Conflict of Interest

The authors declare that the research was conducted in the absence of any commercial or financial relationships that could be construed as a potential conflict of interest.
